# On Quantum Entropy

**DOI:** 10.3390/e24101341

**Published:** 2022-09-23

**Authors:** Davi Geiger, Zvi M. Kedem

**Affiliations:** Courant Institute of Mathematical Sciences, New York University, New York, NY 10012, USA

**Keywords:** quantum entropy, von Neumann entropy, entropic uncertainty principle

## Abstract

Quantum physics, despite its intrinsically probabilistic nature, lacks a definition of entropy fully accounting for the randomness of a quantum state. For example, von Neumann entropy quantifies only the incomplete specification of a quantum state and does not quantify the probabilistic distribution of its observables; it trivially vanishes for pure quantum states. We propose a quantum entropy that quantifies the randomness of a pure quantum state via a conjugate pair of observables/operators forming the quantum phase space. The entropy is dimensionless, it is a relativistic scalar, it is invariant under canonical transformations and under CPT transformations, and its minimum has been established by the entropic uncertainty principle. We expand the entropy to also include mixed states. We show that the entropy is monotonically increasing during a time evolution of coherent states under a Dirac Hamiltonian. However, in a mathematical scenario, when two fermions come closer to each other, each evolving as a coherent state, the total system’s entropy oscillates due to the increasing spatial entanglement. We hypothesize an entropy law governing physical systems whereby the entropy of a closed system never decreases, implying a time arrow for particle physics. We then explore the possibility that as the oscillations of the entropy must by the law be barred in quantum physics, potential entropy oscillations trigger annihilation and creation of particles.

## 1. Introduction

The concept of entropy has been useful in classical physics but extending it to quantum mechanics (QM) has been challenging. In classical physics Boltzmann entropy and Gibbs entropy and their respective H-theorems [1] are formulated in the classical phase space, capturing the practical limitations of specifying the degrees of freedom (DOFs) of a classical state by describing it with randomness. Naturally, in quantum physics the DOFs specify a quantum state. Von Neumann entropy, analogously to the entropy in classical physics, quantifies the randomness of specifying the quantum state, expressed by the classical statistical coefficients of a mixture of quantum states.

Our goal for defining a quantum entropy is to quantify both (i) the inherit randomness of the observables and (ii) the randomness due to the limitations of specifying the DOFs of the quantum state. Our interest in entropy is to better understand the dynamics of quantum information and its impact in physics.

Quantum entropy is not an observable as there is no entropy operator, instead, entropy is a scalar function associated with a state. Thus, we also require quantum entropy to be a scalar invariant under special relativity, canonical transformations of coordinates, and CPT transformations.

We propose a definition of entropy in quantum phase spaces that satisfies those conditions. Quantum entropy has two components. One component is the coordinate-entropy, defined in the phase space of position and momentum. The other component is the spin entropy, which we study elsewhere [2]. Here we focus on this coordinate-entropy of position and momentum and its time evolution, and our study is applicable to both QM and Quantum Field Theory (QFT).

### 1.1. Related Work

Von Neumann entropy [3] captures the randomness associated with not-knowing precisely the quantum state, but does not capture the randomness associated with the observables. Thus, it requires the existence of classical statistics elements (mixed states) in order not to vanish. Wehrl entropy [4] is based on Husimi’s [5] quasiprobability distribution, rooted in projecting states to an overcomplete basis representation of coherent states. These quasiprobability distributions are not relativistic invariant. Note that no two coherent states are orthogonal to each other. Therefore, the Kolmogorov third axiom for a probability distribution, requiring that elementary events be mutually exclusive, is not satisfied. Consequently some probability properties, such as the monotonicity of probabilities and the complement rule, are not satisfied by Husimi’s quasiprobability distribution. These limitations prevent Wehrl entropy from correctly counting the random values of the observables. For example, a quantum state where the projection in position space is a Dirac delta function at $x_{0}$ produces a non-zero value distribution for all possible position coordinates *x* in the classical phase space coordinate (x,p), where *p* is the momentum coordinate. Clearly, this is not the description of the random position *x* in QM. Indeed Wehrl [6] referred to his proposed entropy as a classical entropy for the classical phase space.

The entropic uncertainty principle (EUP) [7,8,9,10] is an extension of the standard uncertainty principle [11,12] for a conjugate pair of observables, where the product of the variances of each observable is replaced by the sum of the entropies for each observable. It is a static statement. We view the sum of these two entropies as a single entropy in phase space, which evolves over time, and the EUP as a lower bound for our proposed entropy for pure states.

Works on quantum thermalization [13,14,15,16] and their references, suggest consideration of a quantum system as a bipartite set of environment states and a subsystem of interest and application of the von Neumann procedure of tracing out the density matrix for the environment states. Then, these works establish a relation between the von Neumann reduced density matrix of a subsystem of interest and the classical entropy. We argue that a complete quantification of randomness of the system, including the randomness of the observables, will lead to a more accurate understanding of the role of entropy in physics.

### 1.2. Our Contribution

Our starting points are pure states, where the DOFs associated with position and spin are precisely specified. We investigate the inherent quantum randomness associated with the observables. This randomness is fully captured by two conjugate pairs of observables satisfying the uncertainty principle [11,12], one associated with space and momentum, and the other associated with the internal spin state. The extension of the uncertainty principle to the entropic uncertainty principle (EUP) [7,8,9,10] suggests that the two entropy components associated with position and momentum play a role in physics. We propose a definition of entropy associated with a quantum pure state, and refer to it as coordinate-entropy. Furthermore, we extend the coordinate-entropy to mix states.

We study the coordinate-entropy’s time evolution for some physical systems, including a coherent state evolution through a potential free Dirac equation, and the hydrogen atom in an excited state transitioning to the ground state with a photon emission. In these scenarios the entropy increases. We also study a collision of two spinless particles. As they come close with each other, due to the superposition of the position wave functions and conservation laws, only the annihilation of these particles and the creation of new particles can prevent the entropy from decreasing. In the process of this analysis, we propose a property of the time evolution of the entropy associated with a potential free Dirac equation.

We then hypothesize an entropy law, universally applicable to particle physics, stating that in a closed physical system the entropy never decreases. The motivation is that the inverse of the amount of randomness, information, cannot be gained in a closed system. Such a law implies irreversibility of time for all physical scenarios where entropy does not stay constant. We complete the paper examining the consequences of such a law in physics.

## 2. Quantum Entropy in Quantum Phase Spaces

We now proceed to define a quantum entropy. An entropy is required to account for both types of DOFs: the coordinate DOFs and the internal DOFs (spin). It must quantify accurately all the randomness associated with the observables of a quantum state. Thus, the probability distributions that define the quantum entropy should express the uncertainty relations of the observables. Moreover, a quantum entropy must be invariant under (i) special relativity transformations, (ii) canonical transformations, and (iii) CPT transformations. Also when considering quantum mixed states, the quantum entropy must also quantify all the randomness associated with the specification of a quantum state.

We first address the coordinate-entropy associated with the coordinate DOFs. Then we mention briefly the spin-entropy associated the spin DOFs, as such study is developed elsewhere [2].

### 2.1. Coordinate-Entropy

We associate with a state |ψ〉 and its density operator ρ=|ψ〉〈ψ| their projection onto the QM eigenstates |x〉 and |p〉 of the operators $\hat{x}$ and $\hat{p}$, respectively. Either one projection, ψ(x)=〈x|ψ〉 or ϕ(p)=〈p|ψ〉, is sufficient to recover the other one via a Fourier transform. However, to account for the randomness of the observables, both |ψ〉 and |ϕ〉, are needed. The quantum coordinate phase space is defined by projecting the density operator to obtain the probability densities $\rho_{x}\left(x\right)=\langle x|\rho |x\rangle ={\left|\psi \left(x\right)\right|}^{2}$ and $\rho_{p}\left(p\right)=\langle p|\rho |p\rangle ={\left|\tilde{\phi }\left(p\right)\right|}^{2}$.

A time evolution of a density function ρ according to a Hermitian Hamiltonian *H* is described by $\rho_{t}=e^{-i\frac{H}{\hbar }t}\;\rho e^{i\frac{H}{\hbar }t}$ and so the evolution of a state in the quantum coordinate phase space is given by the pair of probability densities $\rho_{x}\left(x,t\right)=\langle x|\rho_{t}|x\rangle ={\left|\psi \left(x,t\right)\right|}^{2}$ and $\rho_{p}\left(p,t\right)=\langle p|\rho_{t}|p\rangle ={\left|\tilde{\phi }\left(p,t\right)\right|}^{2}$.

Our formulation of the coordinate-entropy is motivated by previous work including Boltzmann and Gibbs [1], Shanon [17], Jaynes [18], von Neumann entropy, Wehrl entropy, and it amounts to the sum of the two entropies in the EUP. More precisely, let the entropy associated only with the spatial coordinates be the differential entropy Sx=−∫ρx(x,t)lnρx(x,t)(d3x. Let $k=\frac{1}{\hbar }p$ be the spatial frequency (Fourier conjugate of *x*), $\rho_{k}\left(k,t\right)=\hbar^{3}\rho_{p}\left(p,t\right)$ the associated probability density, and Sk=−∫ρk(k,t)lnρk(k,t)(d3k. Then we define the entropy associated with the quantum coordinate phase space distributions as

(1) S=−∫ρ(x,k,t)lnρ(x,k,t)(d3x(d3k=Sx+Sk,

where $\rho \left(x,k,t\right)=\rho_{x}\left(x,t\right)\rho_{k}\left(k,t\right)$. The entropy is dimensionless and thus, invariant under changes of the units of measurements.

A natural extension of this entropy to an *N*-particle QM system is

S=−∫(d3x1(d3k1⋯(d3xN(d3kNρx(x1,⋯,xN,t)ρk(k1,⋯,kN,t)×lnρx(x1,⋯,xN,t)ρk(k1,⋯,kN,t),

where $\rho_{x}\left(x_{1},\cdots ,x_{N},t\right)={\left|\psi \left(x_{1},\cdots ,x_{N},t\right)\right|}^{2}$ and $\rho_{k}\left(k_{1},\cdots ,k_{N},t\right)={\left|\phi \left(k_{1},\cdots ,k_{N},t\right)\right|}^{2}$ are defined in QM via the projection of the state ${|\psi_{t}\rangle }^{N}$ of *N* particles (the product of *N* Hilbert spaces) onto $\langle x_{1}|\cdots \langle x_{N}|$ and $\langle k_{1}|\cdots \langle k_{N}|$ coordinate systems.

Fields in QFT are described by the operators Ψ(x,t), where (x,t) is the space-time, and their spatial Frequency transform Φ(k,t). They are written in terms of operators that create and the annihilate particles at a position and time (x,t). A representation for a system of particles is based on Fock states with occupation number $|n_{q_{1}},n_{q_{2}},\cdots ,n_{q_{i}},\cdots n_{q_{K}}\rangle$, where $n_{q_{i}}$ is the number of particles in a QM state $|q_{i}\rangle$. The number of particles in a Fock state is then $N=\sum_{i=1}^{K}n_{q_{i}}$, and a QFT state is described in a Fock space as $|\mathrm{state}\rangle =\sum_{m}\alpha_{m}|n_{q_{1}},n_{q_{2}},\cdots ,n_{q_{i}},\cdots \rangle$, where *m* is an index over the configurations of a Fock state, $\alpha_{m}\in C$, and $1=\sum_{m}{\left|\alpha_{m}\right|}^{2}$. The QFT phase space state associated with an initial |state〉 and quantum fields Ψ(x,t) and Φ(k,t) is then given by $\left(\langle \mathrm{state}|\rho_{x}^{\mathrm{QFT}}\left(x,t\right)|\mathrm{state}\rangle ,\;\langle \mathrm{state}|\rho_{k}^{\mathrm{QFT}}\left(k,t\right)|\mathrm{state}\rangle \right)$, where the probability density operators for the spatial coordinates are

$$\begin{array}{cc} \rho_{x}^{\mathrm{QFT}}\left(x,t\right) & =\Psi^{†}\left(x,t\right)\Psi \left(x,t\right)\;\mathrm{and}\;\rho_{k}^{\mathrm{QFT}}\left(k,t\right)=\Phi^{†}\left(k,t\right)\Phi \left(k,t\right)\;. \end{array}$$

The QFT coordinate-entropy is then described by the same formulae (1), but for QFT $\rho_{x}\left(x,t\right)=\langle \mathrm{state}|\rho_{x}^{\mathrm{QFT}}\left(x,t\right)|\mathrm{state}\rangle$ and $\rho_{k}\left(k,t\right)=\langle \mathrm{state}|\rho_{x}^{\mathrm{QFT}}\left(k,t\right)|\mathrm{state}\rangle$.

The framework used, QM or QFT, will define which probability density operator is being employed and to which kind of state is being applied.

#### 2.1.1. Uniqueness of the Phase Space and QFT

The variable *x* can be thought as the 3D space where a quantum field is defined and $k=\frac{p}{\hbar }$ as the spatial frequency domain where the Fourier of the quantum field is defined. This makes them unique variables in QFT up to canonical transformations, Lorentz transformations, and CPT transformations.

#### 2.1.2. Mixed Quantum States

We now extend the entropy (1) to mixed states using the QM framework. Consider a mixed state formed from m≥2 pure quantum states $|\psi_{j}\rangle ,j=1,\cdots ,m$, defined by the density matrix $\rho^{M}=\sum_{j=1}^{m}\lambda_{j}|\psi_{j}\rangle \langle \psi_{j}|$, where $\lambda_{j}>0$ and $1=\sum_{j=1}^{m}\lambda_{j}$.

Projecting each component of the density matrix onto the quantum coordinate phase space basis yields $\rho_{j}\left(x,k,t\right)=\lambda_{j}\;|\psi_{j}{\left(x,t\right)|}^{2}\;{\left|\phi_{j}\left(k,t\right)\right|}^{2}$, where 1=∑j=1m∫ρj(x,k,t)(d3x(d3k, which account for the observables probabilities as well as the probabilities associated with specifying the quantum state, namely the probabilities $\lambda_{j},j=1,\cdots ,m$. We define the coordinate-entropy associated with mixed states to be

SM,λj=−∑j=1m∫λj|ψj(x,t)|2|ϕj(k,t)|2lnλj|ψj(x,t)|2|ϕj(k,t)|2(d3x(d3k=−∑j=1mλjlnλj+∑j=1mλjSj,

where Sj=−∫|ψj(x,t)|2ln|ψj(x,t)|2(d3x−∫|ϕj(x,t)|2ln|ϕj(x,t)|2(d3k is the entropy of each pure state. This entropy has two terms: the von Neumann entropy ($-\sum_{j=1}^{m}\lambda_{j}\ln\lambda_{j}$) and the average value of the entropies of the observables for each pure state weighted by the mixed coefficients $\lambda_{j}$. Clearly, the proposed entropy is larger than the von Neumann entropy since it captures both type of randomness, the one associated with the DOFs of a quantum state plus the one associated with the observables. This entropy also differs from Wehrl entropy because it is based on a probability distribution of the observables and not on a quasiprobability distribution that lacks probability properties needed to characterize precisely the randomness of the observables.

When one is interested in quantifying just the randomness of the observables, then one must consider the probability densities $\rho_{r}^{M}\left(x,t\right)=\langle x|\rho^{M}|x\rangle =\sum_{j=1}^{m}\lambda_{j}{\left|\psi_{j}\left(x,t\right)\right|}^{2}$ and $\rho_{k}^{M}\left(k,t\right)=\langle k|\rho^{M}|k\rangle =\sum_{j=1}^{m}\lambda_{j}{\left|\phi_{j}\left(k,t\right)\right|}^{2}$.

In this article we will focus on pure quantum states only as this is the setting of the main new contribution.

### 2.2. Spin-Entropy

It is not possible to know simultaneously the spin of a particle in all three dimensional directions, and this uncertainty, or randomness, was exploited in the Stern–Gerlach experiment [19] to demonstrate the quantum nature of the spin. We explore elsewhere [2] the entropy associated with the quantum spin phase space.

## 3. Entropy Invariant Properties

We now address invariant properties that the coordinate-entropy must satisfy to be considered a physical quantity of interest, namely it must be invariant under canonical transformations, under CPT transformation, and under Lorentz transformation.

QFT is constructed to be invariant under Lorentz transformation, e.g., see [20]. We then adopt the QFT framework for proving the entropy invariance as listed above.

### 3.1. Canonical Transformations

In classical physics canonical transformations are studied for phase spaces mapping $\left(x,k,t\right)\mapsto \left(x^{\prime },k^{\prime },t\right)$ that preserves the form of Hamilton’s equations. We adopt the QFT description of canonical transformations, where the phase space coordinates (x,k) are conjugate variables and not operators.

**Theorem** **1.** *Consider a canonical transformation of coordinates $F:\left(x,k,t\right)\mapsto \left(x^{\prime },k^{\prime },t\right)$. The entropy is invariant under canonical transformations.*

**Proof.**  Let S be the entropy in phase-space relative to a conjugate Cartesian pair of coordinates (x,k) at time *t*, and $S^{\prime }$ to be the entropy of the the new pair of canonical variables $\left(k^{\prime },x^{\prime }\right)$ at time *t*. The canonical transformations induce new operators $\Psi^{\prime }\left(x^{\prime },t\right)$ and $\Phi^{\prime }\left(k^{\prime },t\right)$ in phase space that must satisfy the property that probabilities in infinitesimal volumes are invariant. Thus,

(2) Ψ′†(x′,t)Ψ′(x′,t)(d3x′=Ψ†(x,t)Ψ(x,t)(d3xandΦ′†(k′,t)Φ′(k′,t)(d3k′=Φ†(p,t)Φ(p,t)(d3p.

Let $J_{F}\left(x,k,t\right)=\frac{\left(x^{\prime },k^{\prime }\right)}{\left(x,k\right)}$ be the jacobian matrix of F(x,k,t). The infinitesimal volume invariance at any time *t* gives (d3x′(d3k′=detJF(x,k,t)(d3x(d3k, and applying it to (2) we get

$$\begin{array}{cc} \Psi^{\prime †}\left(x^{\prime },t\right)\Psi^{\prime }\left(x^{\prime },t\right)\Phi^{\prime †}\left(k^{\prime },t\right)\Phi^{\prime }\left(k^{\prime },t\right) & =\frac{1}{\det J_{F}\left(x,k,t\right)}\Psi^{†}\left(x,t\right)\Psi \left(x,t\right)\Phi^{†}\left(p,t\right)\Phi \left(p,t\right)\;. \end{array}$$

Thus,

Sx′+Sk′=−∫(d3x′(d3k′ρ′(x′,k′,t)lnρ′(x′,k′,t)=Sx+Sk+〈lndetJF(x,k,t)〉ρr,k=Sx+Sk,

since for canonical transformations $\det J_{F}\left(x,k,t\right)=1$. □

A special case of canonical transformations of the coordinates $x\mapsto x^{\prime }$, is known as point transformation. Attempts by [21] to extend it to a quantum mechanics point transformation, where the conjugate variables become conjugate operators, are interesting. However, a large set of classical point transformations cannot yield a quantum point transformation, including the transformation from Cartesian coordinates to a spherical coordinate system [22]. The case of translations is possible, and studied by [23] as quantum reference frames. So now we adopt the QM representation, but it is not difficult to adapt it to a QFT representation. When a quantum reference frame is translated by $x_{0}$ along *x*, the state $|\psi_{t}\rangle$ in the position representation becomes $\psi \left(x-x_{0},t\right)=\langle x-x_{0}|\psi_{t}\rangle =\langle x|{\hat{T}}_{P}\left(-x_{0}\right)|\psi_{t}\rangle$, where ${\hat{T}}_{P}\left(-x_{0}\right)=e^{ix_{0}\frac{1}{\hbar }\hat{P}}$, and where $\hat{P}$ is the momentum operator conjugate to $\hat{X}$. When the reference frame is translated by $p_{0}$ along *p*, the state $|\psi_{t}\rangle$ in the momentum representation becomes $\tilde{\phi }\left(p-p_{0},t\right)=\langle p-p_{0}|\psi_{t}\rangle =\langle p|{\hat{T}}_{X}\left(-p_{0}\right)|\psi_{t}\rangle$, where ${\hat{T}}_{X}\left(-p_{0}\right)=e^{i\frac{1}{\hbar }p_{0}\hat{X}}$, and where $\hat{X}$ is the position operator conjugate to $\hat{P}$.

**Theorem** **2** (Frames of reference)**.** *The entropy of a state is invariant under a change of a quantum reference frame by translations along x and along p.*

**Proof.**  Let $|\psi_{t}\rangle$ be a state and S its entropy. We start by showing that Sx=−∫−∞∞|ψ(x,t)|2ln|ψ(x,t)|2(dx is invariant under two types of translations:

(i) translations along *x* by any $x_{0}$
  Sx+x0=−∫−∞∞|ψ(x+x0,t)|2ln|ψ(x+x0,t)|2(dx=Sx,
  which is verified by changing variables.
(ii) translations along *p* by any $p_{0}$
  ψp0(x,t)=〈x|T^X(p0)|ψt〉=∫−∞∞〈x|T^X(p0)|p〉〈p|ψt〉(dp=∫−∞∞〈x|p+p0〉ϕ˜(p,t)(dp=∫−∞∞12πeix1ℏ(p+p0)ϕ˜(p,t)(dp=ψ(x,t)eix1ℏp0,
  implying $|\psi_{p_{0}}{\left(x,t\right)|}^{2}={\left|\psi \left(x,t\right)\right|}^{2}$.

Similarly, by applying both translations to Sp=−∫−∞∞|ϕ˜(p,t)|2ln|ϕ˜(p,t)|2(dp we conclude that $S_{p}$ is invariant under them too. Therefore $S=S_{x}+S_{p}-3\ln\hbar$ is invariant under translations in both *x* and *p*. □

### 3.2. CPT Transformations

We will be focusing on fermions, and thus on the Dirac spinors equation, though the results apply to bosons as well. We return to the QFT represenation where the Dirac Hamiltonian is

HD=∫Ψ†(x,t)−iℏγ0γ→·∇+mcγ0Ψ(x,t)(d3x.

A QFT solution Ψ(x,t) satisfies $\left[H^{D},\Psi \left(x,t\right)\right]=-i\hbar \frac{\Psi (x,t)}{t}$ and the *C*, *P*, and *T* symmetries provide new solutions from Ψ(x,t). As usual, $\Psi^{C}\left(x,t\right)=C{\bar{\Psi }}^{T}\left(x,t\right)$, $\Psi^{P}\left(-x,t\right)=P\Psi \left(-x,t\right)$, $\Psi^{T}\left(x,-t\right)=T\Psi^{*}\left(x,-t\right)$, and $\psi^{\mathrm{CPT}}\left(-x,-t\right)=CPT{\bar{\psi }}^{T}\left(-x,-t\right)$. For completeness, we briefly review the three operations, Charge Conjugation, Parity Change, and Time Reversal.

Charge Conjugation transforms particles Ψ(x,t) into antiparticles ${\bar{\Psi }}^{T}\left(x,t\right)={\left(\Psi^{†}\gamma^{0}\right)}^{T}\left(x,t\right)$. As $C\gamma^{\mu }C^{-1}=-\gamma^{\mu T}$, $\Psi^{C}\left(x,t\right)$ is also a solution for the same Hamiltonian. In the standard representation, $C=i\gamma^{2}\gamma^{0}$ up to a phase. Parity Change $P=\gamma^{0}$, up to a sign, effects the transformation x↦−x. Time Reversal effects t↦−t and is carried by the operator $T=T\hat{K}$, where $\hat{K}$ applies conjugation. In the standard representation $T=i\gamma^{1}\gamma^{3}$, up to a phase. For simplicity of notation we will drop for the QFT superscript of the probability density operator in the theorem that now follows.

**Theorem** **3** (Invariance of the entropy under CPT-transformations)**.** Given a quantum field operator Ψ(x,t), its Fourier transform Φ(k,t), and its entropy $S_{t}$ associated with any initial state, the entropies of $\Psi^{*}\left(x,t\right)$, $\Psi^{P}\left(-x,t\right)$, $\Psi^{C}\left(x,t\right)$, $\Psi^{T}\left(x,-t\right)$, $\Psi^{\mathrm{CPT}}\left(-x,-t\right)$, and their corresponding Fourier transforms are all equal to $S_{t}$.

**Proof.**  The probability densities of $\Psi^{*}\left(x,t\right)$, $\Psi^{T}\left(x,-t\right)$, $\Psi^{P}\left(-x,t\right)$, $\Psi^{C}\left(x,t\right)$, and $\Psi^{\mathrm{CPT}}\left(-x,-t\right)$ are

(3) $$\begin{array}{cc} \rho_{x}^{\mathrm{QFT},\;*}\left(x,t\right) & =\Psi^{T}\left(x,t\right)\Psi^{*}\left(x,t\right)=\Psi^{†}\left(x,t\right)\Psi \left(x,t\right)=\rho^{\mathrm{QFT}}\left(x,t\right)\;,\\ \rho_{x}^{\mathrm{QFT},\;C}\left(x,t\right) & ={\left({\bar{\Psi }}^{T}\right)}^{†}\left(x,t\right)C^{†}C{\bar{\Psi }}^{T}\left(x,t\right)={\bar{\Psi }}^{*}\left(x,t\right){\bar{\Psi }}^{T}\left(x,t\right)=\rho_{x}^{\mathrm{QFT}}\left(x,t\right)\;,\\ \rho_{x}^{\mathrm{QFT},\;P}\left(-x,t\right) & =\Psi^{†}\left(x,t\right){\left(\gamma^{0}\right)}^{†}\gamma^{0}\Psi \left(x,t\right)=\Psi^{†}\left(x,t\right)\Psi \left(x,t\right)=\rho_{x}^{\mathrm{QFT}}\left(x,t\right)\;,\\ \rho_{x}^{\mathrm{QFT},\;T}\left(x,-t\right) & =\Psi^{T}\left(x,t\right)T^{†}T\Psi^{*}\left(x,t\right)=\Psi^{T}\left(x,t\right)\Psi^{*}\left(x,t\right)=\rho_{x}^{\mathrm{QFT}}\left(x,t\right)\;,\\ \rho_{x}^{\mathrm{QFT},\;\mathrm{CPT}}\left(-x,-t\right) & ={\left({\bar{\Psi }}^{T}\right)}^{†}\left(x,t\right){\left(CPT\right)}^{†}\left(CPT\right){\bar{\Psi }}^{T}\left(x,t\right)=\rho_{x}^{\mathrm{QFT}}\left(x,t\right)\;. \end{array}$$

As the operator densities are equal, so are the associated entropies for any given initial state.

Equation (3) also hold for Φ(k,t) and its density. Thus, both entropies terms in $S_{t}=S_{r}+S_{k}$ are invariant under all CPT transformations. □

### 3.3. Lorentz Transformations

**Theorem** **4.** *The entropy is invariant under Lorentz Transformations.*

**Proof.**  The probability elements (dP(x,t)=ρx(x,t)(d3x and (dP(k,t)=ρk(k,t)(d3k are invariant under Lorentz transformations because event probabilities do not depend on the frame of reference. Consider a slice of the phase space with frequency $\omega_{k}=\sqrt{k^{2}c^{2}+{\left(\frac{mc^{2}}{\hbar }\right)}^{2}}$. The volume elements 1ωk(d3k and ωk(d3x, are invariant under the Lorentz group [20], that is, 1ωk(d3k=1ωk′(d3k′ and ωk(d3x=ωk′(d3x′, implying (dV=(d3k(d3x=(d3k′(d3x′=(dV′, where $x^{\prime }$, $k^{\prime },$ and $\omega_{k^{\prime }}$ result from applying a Lorentz transformation to *x*, *k*, and $\omega_{k}$. Thus, from the probability-invariant elements we conclude that $\frac{1}{\omega_{k}}\rho_{x}\left(x,t\right)$ and $\omega_{k}\rho_{k}\left(k,t\right)$ are also invariant under the group. Thus, the phase space density $\rho_{x}\left(x,t\right)\rho_{k}\left(k,t\right)$ is an invariant under Lorentz transformations. Therefore, the entropy is a relativistic scalar. □

Note that in QFT, one scales the operator Φ(k,t) by $\sqrt{2\omega_{k}}$, that is, one scales the creation and the annihilation operators $\alpha^{†}\left(k\right)=\sqrt{\omega_{k}}a^{†}\left(k\right)$ and $\alpha \left(k\right)=\sqrt{\omega }a\left(k\right)$. In this way, the density operator $\Phi^{†}\left(k,t\right)\Phi \left(k,t\right)$ scales with $\omega_{k}$ and becomes a relativistic scalar. Also, with such a scaling, the infinitesimal probability of finding a particle with momentum p=ℏk in the original reference frame is invariant under the Lorentz transformation, though it would be found with momentum $p^{\prime }=\hbar k^{\prime }$.

## 4. The Minimum Entropy Value

The third law of thermodynamics establishes 0 as the minimum classical entropy. However, the minimum of the quantum entropy must be positive due to the uncertainty principle’s lower bound. Let θ(x) be 1 for positive *x* and 0 elsewhere.

**Theorem** **5.** *The minimum entropy of a particle with spin s is 3(1+lnπ)+θ(s)ln2π.*

**Proof.**  The entropy is the sum of the coordinate-entropy and the spin-entropy. The coordinate-entropy (1) is $S_{x}+S_{k}$. Due to the entropic uncertainty principle $S_{x}+S_{k}\geq 3\ln e\pi$ as shown in [7,8,10], with $S_{k}=S_{p}-3\ln\hbar$. To complete the proof, by [2], the minimum spin-entropy is θ(s)ln2π. □

Higgs bosons in coherent states have the lowest possible entropy 3(1+lnπ).

The dimensionless element of volume of integration to define the entropy will not contain a particle unless (d3x(d3k≥1, due to the uncertainty principle, and this may be interpreted as a necessity of discretizing the phase space. We note that the minimum entropy of the discretization of (1) is also 3(1+lnπ), as shown in [24].

We point out that coherent states minimize the uncertainty principle, they also minimize the entropic uncertainty principle (and we show it in Section 5.3) and they also minimize Wehrl entropy as shown by Lieb [25].

## 5. Time Evolution of the Entropy

We now introduce a formalism and characterize time evolution behaviors of the entropy.

### 5.1. A Formalism for Entropy Evolution

We introduce the concept of a QCurve to specify a curve (or path) in a Hilbert space parametrized by time. In QM a QCurve is represented by a triple $\left(|\psi_{0}\rangle ,U(t),\delta t\right)$ where $|\psi_{0}\rangle$ is the initial state, $U\left(t\right)=e^{-i\frac{H}{\hbar }t}$ is the evolution operator, and [0,δt] is the time interval of the evolution. Of course, one may also represent the initial state by a triple $\left(\rho_{0},U\left(t\right),\delta t\right)$, where $\rho_{0}=|\psi_{0}\rangle \langle \psi_{0}|$ is the density matrix. Alternatively, we can represent the initial state in the quantum coordinate phase space by $\left(\langle x|\rho_{0}|x\rangle ,\langle k|\rho_{0}|k\rangle \right)$ or $\left(\langle x|\psi_{0}\rangle ,\langle k|\psi_{0}\rangle \right)$. In QFT the unitary evolution may be represented by the initial condition $\left(\Psi_{0}=\Psi \left(x,0\right),\;\Phi_{0}=\Phi \left(k,0\right)\right)$ or by the initial phase space state $\left(\rho_{x}\left(x,0\right)=\langle \mathrm{state}|\rho_{x}^{\mathrm{QFT}}\left(x,0\right)|\mathrm{state}\rangle ,\;\rho_{k}\left(k,0\right)=\langle \mathrm{state}|\rho_{k}^{\mathrm{QFT}}\left(k,0\right)|\mathrm{state}\rangle \right)$.

We will use any of these representations to describe a QCurve as more convenient for manipulations for the problem at hand.

**Definition** **1** (Partition of E)**.** *Let E to be the set of all the QCurves. We define a partition of E based on the entropy evolution into four blocks:*

C: *The set of QCurves for which the entropy is a constant.*
I: *The set of QCurves for which the entropy is increasing, but it is not a constant.*
D: *The set of QCurves for which the entropy is decreasing, but it is not a constant.*
O: *The set of oscillating QCurves, with the entropy strictly increasing in some subinterval of [0,δt] and strictly decreasing in another subinterval of [0,δt].*

Consider stationary states $|\psi_{t}\rangle =|\psi_{E}\rangle e^{-i\omega t}$ with ω=E/ℏ, where *E* is an energy eigenvalue of the Hamiltonian, and $|\psi_{E}\rangle$ is the time-independent eigenstate of the Hamiltonian associated with *E*.

**Theorem** **6.** *All stationary states are in C.*

**Proof.**  Follows from the time invariance of the probabilities $\rho_{t}=|\psi_{t}\rangle \langle \psi_{t}|=|\psi_{E}\rangle \langle \psi_{E}|$. □

### 5.2. Dispersion of a Fermion Hamiltonian

Dirac’s free-particle Hamiltonian in QM [26] is

(4) $$\begin{array}{c} H=-i\hbar \gamma^{0}\vec{\gamma }\;\cdot \;\nabla +mc\gamma^{0}\;. \end{array}$$

It can be diagonalized in the spatial Fourier domain |k〉 basis to obtain

(5) $$\begin{array}{cc} \omega (k) & =\pm c\sqrt{{\left||k|\right|}^{2}+\frac{m^{2}}{\hbar^{2}}c^{2}}\;, \end{array}$$

where ω(k) is the frequency component of the Hamiltonian. We focus on the positive energy solutions and so the group velocity becomes

(6) $$\begin{array}{cc} v_{g}\left(k\right) & =\nabla_{k}\omega \left(k\right)=\frac{\hbar }{m}\frac{k}{\sqrt{1+{\left(\frac{\hbar ||k||}{mc}\right)}^{2}}}\;. \end{array}$$

In (9) we will use the Taylor expansion of (5) up to the second order, thus requiring the Hessian H(k), with the entries

(7) $$\begin{array}{cc} H_{ij}\left(k\right) & =\frac{\partial^{2}\omega \left(k\right)}{\partial k_{i}k_{j}}=\frac{\hbar }{m}{\left(1+{\left(\frac{\hbar ||k||}{mc}\right)}^{2}\right)}^{-\frac{3}{2}}\left[\delta_{i,j}\left(1+{\left(\frac{\hbar ||k||}{mc}\right)}^{2}\right)-\left(\frac{\hbar k_{i}}{mc}\right)\left(\frac{\hbar k_{j}}{mc}\right)\right]\; \end{array}$$

for the positive energy solution. The three (positive) eigenvalues of H(k) are

$$\begin{array}{cc} \lambda_{1} & =\frac{\hbar }{m}{\left(1+{\left(\frac{\hbar ||k||}{mc}\right)}^{2}\right)}^{-\frac{3}{2}}=\hbar \frac{m^{2}}{{\left(m^{2}+\mu^{2}\left(||k||\right)\right)}^{\frac{3}{2}}}\;, \end{array}$$

$$\begin{array}{cc} \lambda_{2,3} & =\frac{\hbar }{m}{\left(1+{\left(\frac{\hbar ||k||}{mc}\right)}^{2}\right)}^{-\frac{1}{2}}=\hbar \frac{1}{(m^{2}+\mu^{2}{\left(||k||)\right)}^{\frac{1}{2}}}\;, \end{array}$$

where μ(||k||)=ℏ||k||/c is the kinetic energy in mass units. The Hessian is positive definite for positive energy, and so it gives a measure of the dispersion of the wave.

We now consider initial solutions that are localized in space, $\psi_{k_{0}}\left(x-x_{0}\right)=\psi_{0}\left(x-x_{0}\right)e^{ik_{0}\;\cdot \;x}$, where $x_{0}$ is the mean value of *x*. Assume that the variance, ∫(d3x(x−x0)2ρx(x), is finite, where $\rho_{x}\left(x\right)={\left|\psi_{0}\left(x\right)\right|}^{2}$. In a Cartesian representation, we can write the initial state in the spatial frequency domain as $\phi_{x_{0}}\left(k-k_{0}\right)=\phi_{0}\left(k-k_{0}\right)e^{-i\left(k-k_{0}\right)\;\cdot \;x_{0}}$, where $\phi_{0}\left(k\right)$ is the Fourier transform of $\psi_{0}\left(x\right)$, and so the variance of $\rho_{k}\left(k\right)={\left|\phi_{x_{0}}\left(k-k_{0}\right)\right|}^{2}$ is also finite, with the mean in the spatial frequency center $k_{0}$.

The time evolution of $\psi_{k_{0}}\left(x-x_{0}\right)$ according a Hamiltonian with a dispersion relation ω(k), and written via the inverse Fourier transform, is

(8) ψk0(x−x0,t)=1(2π)3∫Φx0(k−k0)e−iω(k)teik·x(d3k.

As $\phi_{x_{0}}\left(k-k_{0}\right)$ fades away exponentially from $k=k_{0}$, we expand (5) in a Taylor series and approximate it by

(9) $$\begin{array}{cc} \omega (k) & \approx v_{p}\left(k_{0}\right)\;\cdot \;k_{0}+v_{g}\left(k_{0}\right)\;\cdot \;\left(k-k_{0}\right)+\frac{1}{2}{\left(k-k_{0}\right)}^{T}H\left(k_{0}\right)\left(k-k_{0}\right)\;, \end{array}$$

where $v_{p}\left(k_{0}\right)$, $v_{g}\left(k_{0}\right)$, and $H(k_{0})$ are the phase velocity $\omega (k_{0}){\hat{k}}_{0}/|k_{0}|$, the group velocity (6), and the Hessian (7) of the dispersion relation ω(k), respectively. Then after inserting (9) into (8), we obtain the quantum dispersion transform

(10) $$\begin{array}{cc} \phi_{x_{k_{0}}^{t}}\left(k-k_{0},t\right) & \approx \frac{1}{Z_{k}}e^{-itv_{p}\left(k_{0}\right)\;\cdot \;k_{0}}\Phi_{x_{k_{0}}^{t}}\left(k-k_{0}\right)N\left(k|k_{0},-it^{-1}H^{-1}\left(k_{0}\right)\right)\;,\\ \psi_{k_{0}}\left(x-x_{k_{0}}^{t},t\right) & \approx \frac{1}{Z_{x}}e^{-itv_{p}\left(k_{0}\right)\;\cdot \;k_{0}}\psi_{k_{0}}\left(x-x_{k_{0}}^{t}\right)*N\left(x|x_{k_{0}}^{t},itH\left(k_{0}\right)\right)\;, \end{array}$$

where $x_{k_{0}}^{t}=x_{0}+v_{g}\left(k_{0}\right)t$, $\Phi_{x_{k_{0}}^{t}}\left(k-k_{0}\right)=\phi_{0}\left(k-k_{0}\right)e^{-i\left(k-k_{0}\right)\;\cdot \;x_{k_{0}}^{t}}$, with Fourier transform $\psi_{k_{0}}\left(x-x_{k_{0}}^{t}\right)$; ∗ denotes a convolution, $Z_{r}$ and $Z_{k}$ normalize the amplitudes, and N is a normal distribution. Consequently, $\psi_{k_{0}}\left(x-x_{k_{0}}^{t},t\right)$ is the spatial Fourier transform of $\Phi_{x_{k_{0}}^{t}}\left(k-k_{0},t\right)$.

The probability densities associated with the probability amplitudes in (10) are

(11) $$\begin{array}{cc} \rho_{x}\left(x-x_{k_{0}}^{t},t\right) & =\frac{1}{Z_{x}^{2}}{\left|\psi_{k_{0}}\left(x-x_{k_{0}}^{t}\right)*N\left(x|x_{k_{0}}^{t},itH\left(k_{0}\right)\right)\right|}^{2}\;,\\ \rho_{k}\left(k-k_{0},t\right) & =\frac{1}{Z_{k}^{2}}{\left|\Phi_{x_{k_{0}}^{t}}\left(k-k_{0}\right)\right|}^{2}\;. \end{array}$$

**Lemma** **1** (Dispersion Transform and Reference Frames)**.** *The entropy associated with (11) is equal to the entropy associated with the simplified probability densities*

(12) $$\begin{array}{cc} \rho_{x}^{S}\left(x,t\right) & =\frac{1}{Z^{2}}{\left|\psi_{0}\left(x\right)*N\left(x|0,itH(k_{0})\right)\right|}^{2}\;,\\ \rho_{k}^{S}\left(k,t\right) & =\frac{1}{Z_{k}^{2}}{\left|\Phi_{0}\left(k\right)\right|}^{2}=\rho_{k}^{S}\left(k,t=0\right)\;. \end{array}$$

**Proof.**  Consider (11). If the frame of reference is translating the position by $x_{k_{0}}^{t}=x_{0}+v_{g}\left(k_{0}\right)t$ and the momentum by $\hbar k_{0}$, we get the simplified density functions (12).

Theorem 2 shows that the entropy in position and momentum is invariant under translations of the position *x* and the spatial frequency *k*, and that completes the proof. □

The time invariance of the density $\rho_{k}^{S}\left(k,t\right)$, and therefore of $S_{k}$, reflects the conservation law of momentum for free particles.

### 5.3. The Coordinate-Entropy of Coherent States Increases with Time

Coherent states, represented by state |α〉, are eigenstates of the annihilator operator. The 1D quantum phase space of observables (x,p) can be constructed by the unitary operator $U\left(x_{0},p_{0}\right)=e^{\frac{i}{\hbar }\left(x_{0}X-p_{0}P\right)}$ applied to zero-state |x=0,p=0〉, that is, they can be constructed as $|\alpha \rangle =|x_{0},p_{0}\rangle =e^{\frac{i}{\hbar }\left(x_{0}X-p_{0}P\right)}|0,0\rangle$, where $\alpha =x_{0}+ip_{0}$. Projecting the state to position space yields $\psi_{\alpha }\left(x\right)=\langle x|\alpha \rangle =e^{-\frac{p_{0}^{2}}{2}}e^{-\frac{1}{2}{\left(x-\sqrt{2}\alpha \right)}^{2}}/\pi^{\frac{1}{4}}$, where $\alpha =\left(x_{0}+ip_{0}\right)/\sqrt{2}$. Squeeze states extend coherent states to all eigenstate solutions of the annihilator operator by allowing different variances to the Gaussian solution, and together their representation in 3D position and momentum space are

(13) $$\begin{array}{cc} \psi_{k_{0}}\left(x-x_{0}\right) & =\langle x|\alpha \rangle =\frac{1}{2^{3}\pi^{\frac{3}{2}}{\left(\det\Sigma \right)}^{\frac{1}{2}}}N\left(x|x_{0},\Sigma \right)e^{ik_{0}\;\cdot \;x}\;,\\ \Phi_{x_{0}}\left(k-k_{0}\right) & =\langle k|\alpha \rangle =\frac{1}{2^{3}\pi^{\frac{3}{2}}{\left(\det\Sigma^{-1}\right)}^{\frac{1}{2}}}N\left(k|k_{0},\Sigma^{-1}\right)e^{i\left(k-k_{0}\right)\;\cdot \;x_{0}}\;, \end{array}$$

where Σ is the spatial covariance matrix.

**Theorem** **7.** *A QCurve with an initial coherent state (13) and evolving according to (4) is in I.*

**Proof.**  To describe the evolution of the initial states (13), we apply (10). Then, after applying Lemma 1,

$$\begin{array}{cc} \rho_{x}^{S}\left(x,t\right) & =\frac{1}{Z_{2}^{2}}N\left(x|0,\Sigma +itH(k_{0})\right)N\left(x|0,\Sigma -itH(k_{0})\right)=N\left(x|0,\frac{1}{2}\Sigma \left(t\right)\right)\;,\\ \rho_{k}^{S}\left(k,t\right) & =N\left(k|0,\Sigma^{-1}\right)\;, \end{array}$$

where $\Sigma \left(t\right)=\Sigma +t^{2}H\left(k_{0}\right)\Sigma^{-1}H\left(k_{0}\right)$. Then

S=Sx+Sk=−∫Nx∣0,12Σ(t)lnNx∣0,12Σ(t)(d3x−∫Nk∣0,Σ−1lnNk∣0,2Σ−1(d3k=3(1+lnπ)+12lndetI+t2(Σ−1H(k0))2.

As $\det\left(I+t^{2}{\left(\Sigma^{-1}H\left(k_{0}\right)\right)}^{2}\right)>0$, the entropy increases over time. □

The theorem suggests that quantum physics has an inherent mechanism to increase entropy for free particles, due to the spatial dispersion property of the Hamiltonian. Note that at t=0 a coherent state (13) reaches the minimum possible coordinate-entropy value.

The dispersion properties of the Dirac and Schrödinger Hamiltonian equations have been studied in the past, and are already present in Feynmann path integral formulation for the free particle [27], where an analytical solution is derived showing the dispersion of the initial localized particle.

### 5.4. A Conjecture on Entropy Evolution

**Conjecture** **1.** *For every single fermion state ${|\Psi \rangle }_{0}$ in Hilbert space, that is evolving under the free fermion Hamiltonian, there exist a finite time parameter T such that ∀t>T the coordinate-entropy will not decrease.*

We present a motivation for this conjecture. The dispersion relation ω(k) for the free fermion Hamiltonian has a positive Hessian (7). Note that Schrödinger Hamiltonian also has a positive Hessian. We also observe some mathematical scenarios where the entropy can decrease temporarily. Given a fermion in a coherent state solution $|\Psi_{0}\rangle$ evolving backwards for a time period *T*. It will yield a solution $|\Psi_{-T}\rangle =e^{i\frac{H}{\hbar }T}|\Psi_{0}\rangle$, with larger entropy than $|\Psi_{0}\rangle$. Then a starting solution $|\Psi_{-T}\rangle$ will evolve forward for a period t∈(0,T], until it reaches back to ${|\Psi \rangle }_{0}$, with entropy decreasing. However, for t>T the entropy of the evolution will increase forever. We discuss this scenario next in Section 5.5. Another physical scenario where the entropy can decrease for a period *T* is when the initial state is a sum of two coherent states, $\langle x|\Psi_{0}\rangle =\Psi_{k_{0}}\left(x-x_{0}\right)+\Psi_{-k_{0}}\left(x+x_{0}\right)$, that is, these parameters model two components away from each other and moving towards each other. For large distances $2x_{0}$, where the overlap of the two components is negligible, the entropy evolution of each component increases due to dispersion, and thus, the entropy increases. As the two components come closer to each other, the overlap increases, and the final probability contain a significant term from the interference of the components. Then, due to the interference, the entropy can decrease. Continuing the evolution, as the two components “pass through each other” and start to move away from each other, the entropy will again start to increase, and will increase forever. The parameter *T* in this case represents the period of a large overlap between the two components up to when they “pass through each other”.

Note that for physical scenarios where the overlap is large enough for causing the entropy to decrease, there is a possible mechanism in nature, outside of the motion equation, to annihilate such solutions (such particles) and create new particles that satisfy the conservation laws that could have the entropy of the evolution to always increase.

### 5.5. Time Reflection

Consider a time-independent Hamiltonian. We investigate the discrete symmetries C and P, and propose that Time Reversal be augmented with Time Translation, say by δt. We refer to the mapping t↦−t+δt as Time Reflection, because as *t* varies from 0 to δt, $t^{\prime }\left(t\right)=-t+\delta t$ varies as a reflection from δt to 0. We define the Time Reflection quantum field

$$\begin{array}{c} \Psi^{T_{\delta }}\left(x,-t+\delta t\right)=T\Psi \left(x,t\right)=T\Psi^{*}\left(x,t\right)\;. \end{array}$$

Note that in contrast to the case of Time Reversal, $\Psi^{T_{\delta }}\left(x,t^{\prime }\right)=T\Psi \left(x,-t^{\prime }+\delta t\right)$, and the entropies associated with Ψ(x,t) and $\Psi^{T_{\delta }}\left(x,t\right)$ are generally not equal. Thus, an instantaneous Time Reflection transformation will cause entropy changes.

We next consider a composition of the three transformation, Charge Conjugation, Parity Change, and Time Reflection.

**Definition** **2** ($\Psi^{{\mathrm{CPT}}_{\delta }}$)**.** *Let the ${\mathrm{CPT}}_{\delta }$ quantum field be*

(14) $$\begin{array}{cc} \Psi^{{\mathrm{CPT}}_{\delta }}\left(-x,-t+\delta t\right) & =\eta_{\delta }CPT{\bar{\Psi }}^{T}\left(x,t\right)=\eta \gamma^{5}{\left(\Psi^{†}\right)}^{T}\left(x,t\right)\;, \end{array}$$

*where η is the product of the phases of each operation, $\eta_{\delta }$ is the phase of time translation, and $\gamma^{5}=i\gamma^{0}\gamma^{1}\gamma^{2}\gamma^{3}$.*

**Definition** **3** ($Q_{{\mathrm{CPT}}_{\delta }}$)**.** *Let $Q_{{\mathrm{CPT}}_{\delta }}$ be $\left(\psi (x,0),U(t),[0,\delta t]\right)\mapsto \left(\psi^{{\mathrm{CPT}}_{\delta }}\left(-x,0\right),U\left(t\right),\left[0,\delta t\right]\right)$.*

Using (14) we see that,

(15) $$\begin{array}{cc} \psi^{{\mathrm{CPT}}_{\delta }}\left(-x,0\right) & =\eta \gamma^{5}{\left(\Psi^{†}\right)}^{T}\left(x,-0+\delta t\right)=\eta \gamma^{5}{\left(\Psi^{†}\right)}^{T}\left(x,\delta t\right)\;. \end{array}$$

**Theorem** **8** (Time Reflection)**.** *Consider a CPT invariant quantum field theory (QFT) with energy conservation, such as Standard Model or Wightman axiomatic QFT [28]. Let $e_{0}=\left(\Psi (x,0),U(t),[0,\delta t]\right)$ be a QCurve solution to such QFT. Then, $e_{1}=Q_{{\mathrm{CPT}}_{\delta }}\left(e_{0}\right)$ is (i) a solution to such QFT, (ii) if $e_{0}$ is in C, D, O, I then $e_{1}$ is respectively in C, I, O, D, making C, I, O, D reflections of C, D, O, I, respectively.*

**Proof.**  Let $t^{\prime }=-t+\delta t$. The QCurve $e_{1}$ describes the evolution of $\psi^{{\mathrm{CPT}}_{\delta }}\left(-x,t^{\prime }\right)$ during the period [0,δt].

Since $e_{0}$ is a solution to a QFT that is CPT-invariant and time-translation invariant, $e_{1}$ is also a solution to the QFT, proving (i).

The time evolution of $\Psi^{{\mathrm{CPT}}_{\delta }}\left(-x,0\right)$ from 0 to δt is described by $\Psi^{{\mathrm{CPT}}_{\delta }}\left(-x,t^{\prime }\right)$, and by (15) $\Psi^{{\mathrm{CPT}}_{\delta }}\left(-x,t^{\prime }\right)=\eta \gamma^{5}{\left(\Psi^{†}\right)}^{T}\left(x,-t^{\prime }+\delta t\right)=\eta \gamma^{5}\Psi^{*}\left(x,\delta t-t^{\prime }\right)$. Thus by Theorem 3, the evolution of $\Psi^{{\mathrm{CPT}}_{\delta }}\left(-x,t^{\prime }\right)$ as $t^{\prime }$ evolves from 0 to δt has the same entropies as $\Psi (x,\delta t-t^{\prime })$. Since $\Psi (x,\delta t-t^{\prime })$ traverses the same path as $\Psi (x,t^{\prime })$ but in the opposite time direction, we conclude that $e_{1}$ produces the time evolution states $\Psi^{{\mathrm{CPT}}_{\delta }}\left(-x,t^{\prime }\right)$ in the time interval [0,δt] traversing the same path and with the same entropies as $\Psi (x,t^{\prime })$, but in the opposite time directions.

Applying the above to a QCurve respectively in I, D, C, O, results in a QCurve respectively in D, I, C, O, proving (ii). □

**Figure 1:**
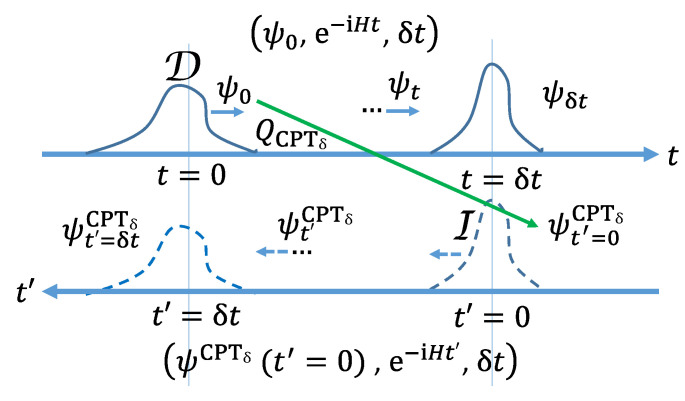
A visualization of the Time Reflection Theorem. (i) Axis *t*: A QCurve $e_{1}=\left(\Psi_{0}=\Psi \left(x,0\right),e^{-iHt},\delta t\right)$. (ii) Axis $t^{\prime }=\delta t-t$: The antiparticle QCurve is created as $e_{2}=Q_{{\mathrm{CPT}}_{\delta }}\left(e_{1}\right)=\left(\Psi^{{\mathrm{CPT}}_{\delta }}\left(-x,t^{\prime }=0\right),e^{-iHt^{\prime }},\delta t\right)$. Axis $t^{\prime }$ shows the evolution as going forward in time $t^{\prime }$. The evolution of $\Psi^{{\mathrm{CPT}}_{\delta }}\left(-x,t^{\prime }\right)=\eta \gamma^{5}{\left(\Psi^{†}\right)}^{T}\left(x,\delta t-t^{\prime }\right)$ is mirroring the evolution of Ψ(x,t), with $t=t^{\prime }$ evolving from 0 to δt. If $e_{1}\in D$, then $e_{2}\in I$.

For a visualization see Figure 1.

### 5.6. Entropy Oscillations

Consider a Hamiltonian $H^{\prime }=H+H^{I}$, where $|H^{I}\left|\ll |H\right|$ accounts for additional interactions, and the initial eigenstate $|\psi_{E_{i}}\rangle$ of *H* associated with the eigenvalue $E_{i}=\hbar \omega_{i}$. The time evolution of $|\psi_{E_{i}}\rangle$ is

$$|\psi_{t}\rangle =e^{-i\frac{\left(H+H^{I}\right)}{\hbar }t}|\psi_{E_{i}}\rangle =\sum_{k=1}^{n}\alpha_{k}\left(t\right)|\psi_{E_{k}}\rangle \;,$$

where *n* is the number of the eigenvectors of *H*. Fermi’s golden rule [29,30] approximates the coefficients of transition for k≠i and short time intervals by

$$\begin{array}{cc} \alpha_{k}\left(t\right) & \approx \frac{H_{i,k}^{I}}{\hbar (\omega_{i}-\omega_{k})}\left(-2\sin^{2}\left(\frac{(\omega_{i}-\omega_{k})t}{2}\right)+i\sin\left((\omega_{i}-\omega_{k})t\right)\right)\;. \end{array}$$

**Theorem** **9** (Entropy Oscillations)**.** *Consider the QCurve $\left(|\psi_{E_{i}}\rangle ,U\left(t\right)=e^{-i\frac{\left(H+H^{I}\right)}{\hbar }t},T\right)$ with $\hbar \omega_{1}$ the ground state value of H and $T=\frac{2\pi }{|\omega_{i}-\omega_{1}|}$. Assume that $|\alpha_{1}{\left(t\right)|}^{2},|\alpha_{i}{\left(t\right)|}^{2}\gg {\left|\alpha_{k}\left(t\right)\right|}^{2}$ for k≠1,i and t∈[0,T]. Then the QCurve is in O.*

**Proof.**  With the theorem’s assumptions, we can approximate the position and the momentum probability densities associated with $|\psi_{t}\rangle$ by

$$\begin{array}{cc} \rho_{x}\left(x,t\right) & \approx {\left|\sqrt{1-|\alpha_{1}{\left(t\right)|}^{2}}\langle x|\psi_{E_{i}}\rangle +\alpha_{1}\left(t\right)\langle x|\psi_{E_{1}}\rangle \right|}^{2}\;,\\ \rho_{k}\left(k,t\right) & \approx {\left|\sqrt{1-|\alpha_{1}{\left(t\right)|}^{2}}\langle k|\psi_{E_{i}}\rangle +\alpha_{1}\left(t\right)\langle k|\psi_{E_{1}}\rangle \right|}^{2}\;. \end{array}$$

The time coefficients of $\rho_{x}\left(x,t\right)$ and $\rho_{k}\left(k,t\right)$ are the same, and they all return to the same values simultaneously after a period of *T*, and so the entropy will return to its previous value too. As the entropy is not a constant, it must be oscillating. □

Thus, when Fermi’s golden rule can be applied, the coefficients of the transition probabilities of the unitary evolution of a state oscillate, and the entropy associated with the evolution of such a state will also oscillate with the same period.

**Theorem** **10** (Coefficients for two states)**.** *Consider a particle in an eigenstate $|\psi_{E_{1}}\rangle$ of a Hamiltonian H that has only two eigenstates $|\psi_{E_{1}}\rangle$ and $|\psi_{E_{2}}\rangle$ with eigenvalues $E_{1}=\hbar \omega_{1}$ and $E_{2}=\hbar \omega_{2}$, respectively. Let this particle interact with an external field (such as the impact of a Gauge Field), requiring an additional Hamiltonian term $H^{I}$ to describe the evolution of this system.*

*Let $\omega_{i,j}^{I}=\frac{1}{\hbar }\langle \psi_{E_{i}}|H^{I}|\psi_{E_{j}}\rangle$, $\omega_{1}^{\mathrm{total}}=\omega_{1}+\omega_{11}^{I}$, $\omega_{2}^{\mathrm{total}}=\omega_{2}+\omega_{22}^{I}$, $\eta =\sqrt{{\left(\omega_{1}^{\mathrm{total}}-\omega_{2}^{\mathrm{total}}\right)}^{2}+4{\left(\omega_{12}^{I}\right)}^{2}}$, and $\lambda_{\pm }=\frac{\omega_{1}^{\mathrm{total}}+\omega_{2}^{\mathrm{total}}\pm \eta }{2}$. Then, the probability of the particle to be in state $|\psi_{E_{2}}\rangle$ at time t is*

$$\frac{4{\left(\omega_{12}^{I}\right)}^{2}}{\eta^{2}}\sin^{2}\frac{(\lambda_{+}-\lambda_{-})t}{2}\;.$$

**Proof.**  The Hamiltonians in the basis $|\psi_{E_{1}}\rangle ,|\psi_{E_{2}}\rangle$ are

$$\begin{array}{c} H=\hbar \left(\begin{array}{cc} \omega_{1} & 0\\ 0 & \omega_{2} \end{array}\right)\;\mathrm{and}\;H^{I}=\hbar \left(\begin{array}{cc} \omega_{11}^{I} & \omega_{12}^{I}\\ \omega_{12}^{I} & \omega_{22}^{I} \end{array}\right)\;, \end{array}$$

where the real values satisfy $\omega_{21}^{I}=\omega_{12}^{I}$ as $H^{I}$ is Hermitian. The eigenvalues of the symmetric matrix $H^{\prime }=H+H^{I}$ are $\hbar \lambda_{\pm }$, and so we can decompose it as

(16) $$\begin{array}{cc} H^{\prime } & =\hbar \left(\begin{array}{cc} \omega_{1}^{\mathrm{total}} & \omega_{12}^{I}\\ \omega_{12}^{I} & \omega_{2}^{\mathrm{total}} \end{array}\right)=\left(\begin{array}{cc} \cos\theta & -\sin\theta \\ \sin\theta & \cos\theta \end{array}\right)\left(\begin{array}{cc} \hbar \lambda_{+} & 0\\ 0 & \hbar \lambda_{-} \end{array}\right)\left(\begin{array}{cc} \cos\theta & \sin\theta \\ -\sin\theta & \cos\theta \end{array}\right)\;, \end{array}$$

where

(17) $$\theta =\frac{1}{2}\arcsin\frac{2\omega_{12}^{I}}{\eta }\;.$$

The time evolution of $|\psi_{E_{1}}\rangle$ is $|\psi_{t}\rangle =e^{-i\frac{\left(H+H^{I}\right)}{\hbar }t}|\psi_{E_{1}}\rangle =\sum_{k=1}^{2}\alpha_{k}\left(t\right)|\psi_{E_{k}}\rangle$, and projecting onto $\langle \psi_{E_{j}}|$, we get $\alpha_{j}\left(t\right)=\langle \psi_{E_{j}}|e^{-i\frac{\left(H+H^{I}\right)}{\hbar }t}|\psi_{E_{1}}\rangle$. From (16),

$$\begin{array}{cc} e^{-i\frac{H^{\prime }}{\hbar }t} & =\left(\begin{array}{cc} \cos\theta & -\sin\theta \\ \sin\theta & \cos\theta \end{array}\right)\left(\begin{array}{cc} e^{-i\lambda_{+}t} & 0\\ 0 & e^{-i\lambda_{-}t} \end{array}\right)\times \left(\begin{array}{cc} \cos\theta & \sin\theta \\ -\sin\theta & \cos\theta \end{array}\right) \end{array}$$

Thus,

$$\begin{array}{cc} \left(\begin{array}{c} \alpha_{1}\left(t\right)\\ \alpha_{2}\left(t\right) \end{array}\right) & =e^{-i\frac{H^{\prime }}{\hbar }t}\left(\begin{array}{c} 1\\ 0 \end{array}\right)=\left(\begin{array}{c} \cos^{2}\theta \;e^{-i\lambda_{+}t}+\sin^{2}\theta \;e^{-i\lambda_{-}t}\\ \sin2\theta \;\left(\frac{e^{-i\lambda_{+}t}-e^{-i\lambda_{-}t}}{2}\right) \end{array}\right)\;, \end{array}$$

and so

$$\begin{array}{cc} \left(\begin{array}{c} |\alpha_{1}{\left(t\right)|}^{2}\\ |\alpha_{2}{\left(t\right)|}^{2} \end{array}\right) & =\left(\begin{array}{c} 1-\frac{1}{2}\sin^{2}2\theta \;\left(1-\cos(\lambda_{-}-\lambda_{+})t\right)\\ \frac{1}{2}\sin^{2}2\theta \;\left(1-\cos(\lambda_{-}-\lambda_{+})t\right) \end{array}\right)\;. \end{array}$$

As $1-\cos\left(\lambda_{-}-\lambda_{+}\right)t=2\sin^{2}\frac{(\lambda_{+}-\lambda_{-})t}{2}$, the probability of being in state $|\psi_{E_{2}}\rangle$ at time *t* is $|\alpha_{2}{\left(t\right)|}^{2}=\sin^{2}2\theta \;\sin^{2}\frac{(\lambda_{+}-\lambda_{-})t}{2}$. Using (17), completes the proof. □

If $\omega_{1}\gg \omega_{11}^{I}$, $\omega_{2}\gg \omega_{22}^{I}$, and $|\omega_{1}-\omega_{2}|\gg \omega_{12}^{I}$, then $\lambda_{+,-}\approx \omega_{1,2}$, and the coefficient of transition becomes $|\alpha_{2}{\left(t\right)|}^{2}\approx \frac{4{\left(\omega_{12}^{I}\right)}^{2}}{{\left(\omega_{1}-\omega_{2}\right)}^{2}}\sin^{2}\frac{(\omega_{2}-\omega_{1})t}{2}$, which is Fermi’s golden rule [29,30].

## 6. Entropy Evolution in Physical Scenarios

We now apply to physical scenarios the formalism developed for characterizing the time evolution of the entropy, including analysis of experiments conducted with particles and atoms.

### 6.1. A Two-Particle Collision

Consider a two-fermions or a two-massive-bosons system

$$\begin{array}{cc} |\psi_{t}\rangle & =\frac{1}{\sqrt{C_{t}}}\left(|\psi_{t}^{1}\rangle |\psi_{t}^{2}\rangle \mp |\psi_{t}^{2}\rangle |\psi_{t}^{1}\rangle \right)\;, \end{array}$$

where $C_{t}$ is the normalization constant that may evolve over time and the signs “∓” represent fermions (“−”) and bosons (“+”). When $|\psi_{t}^{1}\rangle$ and $|\psi_{t}^{2}\rangle$ are orthogonal to each other, $C_{t}=2$. Projecting on $\langle x_{1}|\langle x_{2}|$ and on $\langle k_{1}|\langle k_{2}|$,

$$\begin{array}{cc} \psi (x_{1},x_{2},t) & =\frac{1}{\sqrt{C_{t}}}\left(\psi_{1}\left(x_{1},t\right)\psi_{2}\left(x_{2},t\right)\mp \psi_{1}\left(x_{2},t\right)\psi_{2}\left(x_{1},t\right)\right)\;,\\ \psi (k_{1},k_{2},t) & =\frac{1}{\sqrt{C_{t}}}\left(\phi_{1}\left(k_{1},t\right)\phi_{2}\left(k_{2},t\right)\mp \phi_{1}\left(k_{2},t\right)\phi_{2}\left(k_{1},t\right)\right)\;. \end{array}$$

The entropy of the two-particle system, discarding the spin-entropy which is constant throughout the collision, is then

S|ψt1〉,|ψt2〉=−∫(d3x1∫(d3x2ρx(x1,x2,t)lnρx(x1,x2,t)−∫(d3k1∫(d3k2ρk(k1,k2,t)lnρk(k1,k2,t).

Consider a collision of two particles, each one described by an initial coherent state with position variance $\sigma^{2}$, centered at $c_{1}$ and $c_{2}$, and moving towards each other along the *x*-axis with center momenta $\hbar k_{0}$ and $-\hbar k_{0}$. They can be represented in position and momentum space as

(18) $$\begin{array}{cc} \Psi_{1}\left(x,t\right) & =\frac{e^{-ik_{0}v_{p}\left(k_{0}\right)t}}{Z_{1}}e^{ik_{0}x}N\left(x|c_{1}+v_{g}\left(k_{0}\right)t,\sigma^{2}+itH\left(k_{0}\right)\right)\;,\\ \Psi_{2}\left(x,t\right) & =\frac{e^{-ik_{0}v_{p}\left(k_{0}\right)t}}{Z_{1}}e^{-ik_{0}x}N\left(x|c_{2}-v_{g}\left(k_{0}\right)t,\sigma^{2}+itH\left(-k_{0}\right)\right)\;,\\ \Phi_{1}\left(k,t\right) & =\frac{e^{-itv_{p}\left(k_{0}\right)k_{0}}}{Z_{k_{0}}}e^{i\left(k-k_{0}\right)\left(c_{1}+v_{g}\left(k_{0}\right)t\right)}N\left(k|k_{0},{\left(\sigma^{2}+itH\left(k_{0}\right)\right)}^{-1}\right)\;,\\ \Phi_{2}\left(k,t\right) & =\frac{e^{-itv_{p}\left(k_{0}\right)k_{0}}}{Z_{k_{0}}}e^{i\left(k+k_{0}\right)\left(c_{2}-v_{g}\left(k_{0}\right)t\right)}N\left(k|-k_{0},{\left(\sigma^{2}+itH\left(-k_{0}\right)\right)}^{-1}\right)\;. \end{array}$$

**Figure 2:**
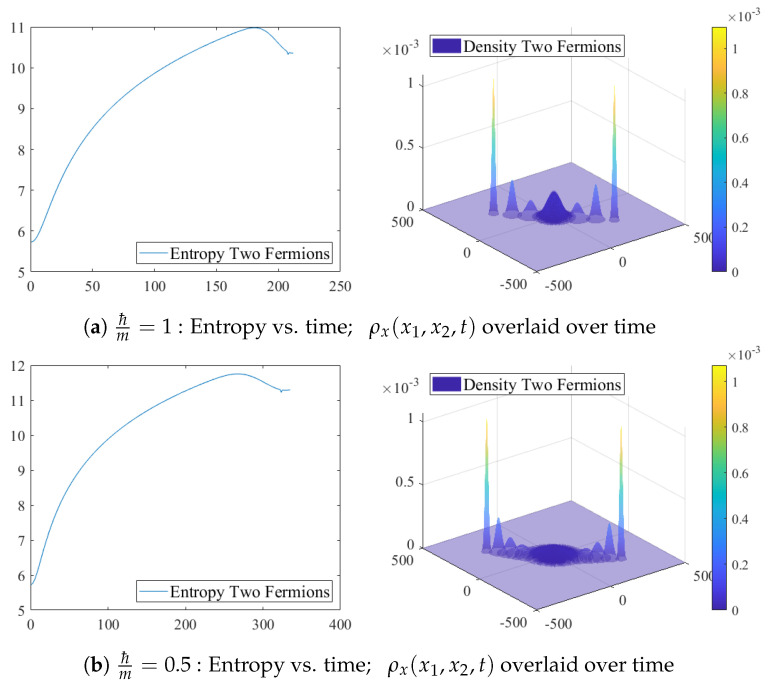
Collision of two fermions with individual amplitudes (18), parameters $k_{0}=1$, $c_{2}=-c_{1}=300$, speed of light c=1, a grid of 1000 points for $x_{1},x_{2},k_{1},k_{2}$. The left column shows entropy vs. time. The right column shows snapshots of the density at initial time, final time, and intervals of 100 time units, overlaid on single plots. The *z*-axis represents the density, and the *x* and *y* axes represent the $x_{1}$ and $x_{2}$ values, respectively. As the particles approach each other, their individual densities disperse, the maximum values are reduced, and the entropy increases. Only when the particles are close to each other, the interference reduces the total entropy.

Figure 2 shows that when the two particles are far apart, the entropy of the system is close to the sum of the two individual entropies, with each one increasing over time. The spatial entanglement decreases the uncertainty, and therefore the entropy too. The competition between the increase of the entropy of the individual particles and the decrease of the entropy due to entanglement results in an oscillation and the decrease in the total entropy when the two particles are close to each other.

### 6.2. The Hydrogen Atom and Photon Emission

The QED Hamiltonian for the hydrogen atom is

$$\begin{array}{cc} H(p,r,q) & =\sum_{i=1}^{3}\frac{{\left(p^{i}-\frac{e}{c}A^{i}\left(q\right)\right)}^{2}}{2m}-\frac{e^{2}}{r}+\sum_{\lambda =1}^{2}\hbar \omega_{q}a_{\lambda }^{†}\left(q\right)a_{\lambda }\left(q\right)\;, \end{array}$$

where the photon’s helicity λ=1,2, $\omega_{q}=\left|q\right|c$, the creation and the annihilation operators of photons satisfy $\left[a_{\lambda }\left(p\right),a_{\lambda^{\prime }}^{†}\left(q\right)\right]=\delta_{\lambda ,\lambda^{\prime }}\delta \left(p-q\right)$, and the electromagnetic vector potential is

$$\begin{array}{cc} {\tilde{A}}^{i}\left(q\right) & =\sqrt{2\pi \hbar c^{2}}\sum_{\lambda =1}^{2}\frac{1}{\sqrt{\omega_{q}}}\left(\epsilon_{\lambda }^{i}\left(q\right)a_{\lambda }\left(q\right)+\epsilon_{\lambda }^{*i}\left(q\right)a_{\lambda }^{†}\left(q\right)\right)\;, \end{array}$$

and in the Coulomb Gauge (∇·A=0), for $q=|q|(\sin\theta_{q}\cos\phi_{q},\sin\theta_{q}\sin\phi_{q},\cos\theta_{q})$, the polarizations satisfy $\epsilon_{1}\left(q\right)=\left(\cos\theta_{q}\cos\phi_{q},\cos\theta_{q}\sin\phi_{q},\sin\theta_{q}\right)$ and $\epsilon_{2}\left(q\right)=\left(-\sin\phi_{q},\cos\phi_{q},0\right)$.

The state of the atom can be described by ${|n,l,m\rangle }_{e^{-}}{|q,\lambda \rangle }_{\gamma }$, where n,l,m are the quantum numbers of the electron $e^{-}$, and *q* and λ are the momentum and the helicity of the photon γ. We next consider the Lyman-alpha transition, |n=2,l=1,m=0〉|0〉→|n=1,l=0,m=0〉|q,λ〉 with the emission of a photon with wavelength λ≈121.567d−9m.

We first evaluate the electron’s entropy at both states |n=2,l=1,m=0〉 and |n=1,l=0,m=0〉. For simplicity, we consider the Schrödinger approximation to describe the electron state with the energy change in this transition of $\Delta E_{n=2\to n=1}\approx \left(1-2^{-2}\right)\times 13.6e\;V=10.2e\;V$. We now compute the difference between the final and the initial state entropy in three steps.

(i) The position probability amplitudes described in [31] and the associated entropies are
  $$\begin{array}{cc} \psi_{2,1,0}\left(\rho ,\theta ,\phi \right) & =\frac{1}{\sqrt{32\pi }}{\left(\frac{1}{a_{0}}\right)}^{\frac{3}{2}}\;\rho e^{-\frac{\rho }{2}}\cos\left(\theta \right)\;\to \;S_{x}\left(\psi_{2,1,0}\right)\approx 6.120+\ln\pi +3\ln a_{0}\;,\\ \psi_{1,0,0}\left(\rho ,\theta ,\phi \right) & =\frac{1}{\sqrt{\pi }}{\left(\frac{1}{a_{0}}\right)}^{\frac{3}{2}}\;e^{-\rho }\;\to \;S_{x}\left(\psi_{1,0,0}\right)\approx 3.000+\ln\pi +3\ln a_{0}\;, \end{array}$$
  where $a_{0}\approx 5.292\times 10^{-11}m$ is the Bohr radius, and $\rho =r/a_{0}$.
(ii) The momentum probability amplitudes described in [31] and the associated entropies are
  $$\begin{array}{cc} \Phi_{2,1,0}\left(p,\theta_{p},\phi_{p}\right) & =\sqrt{\frac{128^{2}}{2\pi p_{0}^{3}}}\;\frac{p}{p_{0}}\;{\left(1+{\left(2\frac{p}{p_{0}}\right)}^{2}\right)}^{-3}\;\cos\left(\theta_{p}\right)\;\to \;S_{p}\left(\Phi_{2,1,0}\right)\approx 0.042+3\ln p_{0}\;,\\ \Phi_{1,0,0}\left(p,\theta_{p},\phi_{p}\right) & =\sqrt{\frac{32}{\pi \;p_{0}^{3}}}{\left(1+{\left(\frac{p}{p_{0}}\right)}^{2}\right)}^{-2}\;\to \;S_{p}\left(\Phi_{1,0,0}\right)\approx 2.422+3\ln p_{0}\;, \end{array}$$
  where $p_{0}=\hbar /a_{0}$.
(iii) Therefore, $\Delta S_{2,1,0\to 1,0,0}=S_{x}\left(\psi_{1,0,0}\right)+S_{p}\left(\Phi_{1,0,0}\right)-S_{x}\left(\psi_{2,1,0}\right)-S_{p}\left(\Phi_{2,1,0}\right)\approx -0.740\;.$

Thus, the entropy of the electron is reduced by approximately 0.740 during the transition |n=2,l=1,m=0〉→|n=1,l=0,m=0〉.

We next evaluate the entropy associated with the randomness in the emission of the photon. Due to energy conservation, the energy must satisfy |q|c≈10.2eV, where *c* is the speed of light. The associated energy uncertainty is very small. The main randomness for the photon is in specifying the direction of the emission. The angular momentum of the electron along *z* (m=0) does not change between |n=2,l=1,m=0〉 and |n=1,l=0,m=0〉. The spin 1 of the photon is along its motion, and conserves the total angular momentum of the system. Thus, to conserve angular momentum along *z*, the photon must be moving perpendicularly to the *z* axis, that is, $\theta_{q}=\frac{\pi }{2}$, and so the polarization vectors must be $\epsilon_{1}\left(q\right)=\left(0,0,1\right)$ and $\epsilon_{2}\left(q\right)=\left(-\sin\phi_{q},\cos\phi_{q},0\right)$. The angle $\phi_{q}$ is completely unknown, with the entropy ln2π. Then we observe that the entropy increases, as

$$\begin{array}{c} \Delta S_{|n=2,l=1,m=0\rangle |0\rangle \;\to \;|n=1,l=0,m=0\rangle |q,\lambda \rangle }\approx \ln2\pi -0.740=1.098\;. \end{array}$$

Consider now an apparent time-reversing scenario in which an apparatus emitted photons with energy $E_{\gamma }=\hbar \left|\omega_{n=2,l=1,m=0}-\omega_{n=1,l=0,m=0}\right|$ to strike a hydrogen atom with its electron in the ground state. The photon had to follow a precise direction towards the atom, and a very small uncertainty in the direction implies low photon entropy. Once the atom absorbs the photon, the energy of the electron in the ground state suffices for a jump into an excited state. The entropy increases again, as the entropy of the excited state is larger than the entropy of the ground state (accounting for the low photon entropy).

#### Experiments in a Reflective Cavity

More recent sophisticated experiments have addressed time reversibility of quantum mechanics, see, e.g., [32,33]. A cavity is created with nearly perfect mirrors and an atom with an electron in an excited state is placed inside the cavity. Then the atom goes to the ground state and a photon is emitted. Then, the cavity mirror reflects the photon, which carries the same phase as the emitted photon. The ground state atom absorbs the photon and the electron jumps back to the excited state, and the process restarts. The whole process is then apparently reversible, meaning it starts with the atom and the excited electron and ends with the atom and the excited electron.

We do not interpret this process as a demonstration of quantum time reversibility. Our reasoning follows. Let $|a^{e}\rangle$ denote the state of the atom with the electron in the excited state, while $|a^{g}\rangle$ denotes the state of the atom in the ground state. At the start of this process the atom state is $|a^{e}\rangle$ and once the electron goes to the ground state and emits a photon the new state is denoted by $|a^{g},\gamma \rangle$, with a photon in the state |γ〉. More generally, the atom can be in a superposition of the two states, $|a^{e}\rangle$ and $|a^{g},\gamma \rangle$. Note that the state $|a^{g},\gamma \rangle$ requires the atom to recoil due to momentum conservation. Since the photon is emitted to a random direction (constrain by the angular momentum conversation) the recoil of the atom must also have such randomness, and the pair of states $|a^{g}\rangle$ and |γ〉 must be entangled by the motion direction variable (see for example [34,35] and references). If one observes the atom recoil direction one will know the direction of the photon emission. Next in the process, the photon is reflected by the cavity. After the photon is reflected, due to momentum conservation, the cavity must now be in a new quantum state, |c〉, carrying twice the momentum the photon had when it was emitted. Since the photon emission direction is a random variable, so is the cavity motion direction. Then the three states $|a^{g}\rangle$, |γ〉 and |c〉 must now be entangled. Observing the momentum of the atom or of the cavity will reveal all other motion directions.

During this process, a flow of information also occurs, that is the entropy associated with the isolated states vary in time. The system evolved from the state $|a^{e}\rangle$, to the entangled state $|a^{g},\gamma \rangle$, then to the entangled state $|a^{g},\gamma ,c\rangle$, and finally, after the atom absorbs the photon, it is in the entangled state $|a^{e},c\rangle$. It is clear that the introduction of the cavity adds another quantum state to the system. For the initial state $|a^{e}\rangle$ the cavity motion was assumed to be zero. For the final state there is an uncertainty in the motion direction of the atom entangled with the motion direction of the cavity. Thus, the final state has a larger entropy than the initial state and the process is not reversible.

## 7. An Entropy Law and a Time Arrow

In classical statistical mechanics, the entropy provides a time arrow through the second law of thermodynamics [36]. We have shown that due to the dispersion property of the fermionic Hamiltonian, some states, such as coherent states, evolve with an increasing entropy. However, current quantum physics is time reversible, and it is possible to have state evolution where the entropy oscillates. This includes the scenario in the hydrogen atom studied earlier, where the excited state of the electron with no photon and the ground state of the electron with a photon emission are two possible states where quantum physics describe an oscillation which we showed leads to the entropy oscillation.

We hypothesize the following

**Law**  (The Entropy Law)**.** *The entropy of an isolated quantum system is an increasing function of time.*

It is an information-theoretic conjecture about isolated quantum states, whereby information (the inverse of the entropy) cannot be gained. We note that it does not require any observer making any measurement.

An evidence for such a law is the hydrogen atom scenario discussed earlier. According to QED, and due to photon fluctuations of the vacuum, the state of an electron in an excited state of the hydrogen atom is in a superposition with the ground state, and the entropy would decrease within a time interval $2\pi /|\omega_{n=2,l=1,m=0}-\omega_{n=1,l=0,m=0}|$. Instead, interrupting the oscillation, the electron jumps to the ground state and a photon is created/emitted, increasing the entropy. We hypothesize that the entropy law is the trigger for the photon creation.

We complete the paper wondering whether, in light of the hypothesized entropy law, all quantum states are indeed always in a superposition of all quantum states evolving according to the unitary evolution dictated by the Hamiltonian of the system as current QM asserts. In this case, no collapse of the wave function exists. Alternatively, and according to the QFT description, the creation and annihilation of particles occur and can interrupt the unitary evolution. In this QFT framework, if entropy oscillation scenarios can occur, e.g., as described by the Fermi Golden Rule transition [29,30], then the entropy law would trigger a collapse of a state to a new state where the entropy will increase during the following evolution. This could possibly describe the emission of the photon when the electron falls to the ground state in the hydrogen atom, or the collision of two particles, creating the new particles. In this case, like in the Copenhagen interpretation of QM, the collapse of the state would occur, but in contrast to the Copenhagen interpretation, it would not require a measurement (or an observer).

## 8. Conclusions

Capturing all the information of a quantum state requires specification of the parameters associated with the DOFs of a quantum state as well as the intrinsic randomness of the quantum state. The intrinsic randomness is associated with a conjugate pair of observables, satisfying the uncertainty principle. We proposed a coordinate-entropy defined in the quantum phase spaces, the space of all possible states projected in the Fourier conjugate basis of position, and spatial frequency. Even though these observables are the same variables used in the classical entropy, the motivation and quantification are quite different. For the classical case, the randomness originates in the practical difficulties in specifying the DOFs precisely, while for the quantum pure state case, the randomness is due to the intrinsic quantum state observables characterized by a pair of conjugate observables that satisfy the uncertainty principle.

This definition of the coordinate-entropy and quantum phase spaces possesses desirable properties, including invariance under canonical transformations, under Lorentz trasnformations, and under CPT transformations. We extended this entropy for the more general case where there is a randomness associated with specifying the quantum state, leading to a mixed quantum state. For mixed states, the entropy is always larger than von Neumann entropy due to the accounting for the randomness associated with the observables of each pure state.

We analyzed the entropy evolution through the partition of QCurves into the four sets C, I, O, D. We showed that the Dirac’s Hamiltonian disperses information due to its positive Hessian, causing coherent states time evolution to increase entropy. We proved that Time Reflection transforms QCurves in C, I, O, D into QCurves in C, D, O, I, respectively. We proved that an initial eigenstate of a Hamiltonian evolving with the addition of a Hamiltonian term not only causes a state oscillation (as suggested by Fermi’s golden rule when the appropriate approximations hold) but also causes entropy oscillation. We showed that the entropy increases when an electron in an excited state of the hydrogen atom falls to the ground state emitting a photon. We also showed that experiments with near perfect cavity with atoms in excited states do not describe reversible processes, but rather processes such that the information of the entanglement of the atom with a cavity motion direction cannot be neglected. We studied collisions of two particles, each evolving as a coherent state, and showed that as they come closer to each other the total system’s entropy oscillates.

We hypothesized an entropy law that the entropy of a closed quantum system increases with time. The motivation for the law is that information (inverse of the amount of randomness) cannot increase in a closed quantum system. This law implies the irreversibility of time for scenarios where the entropy is not constant.

The results are applicable to both the Quantum Mechanics (QM) and the Quantum Field Theory (QFT) settings, but we generally presented them in the more convenient setting.

For the oscillation scenarios, the entropy law triggers the collapse of a state to a new state where the new evolution will cause the entropy to increase. Such a collapse is accompanied by particle creation or annihilation. In this case, the entropy law determines that the event of particle creation and/or annihilation does occur, regardless of an observer performing a measurement. In this view, a measurement is a physical process that activates the hypothesized entropy law. Thus, for example, the phenomena described by the double slit experiment would imply that, at the sensors screen, the absorption (annihilation) of the particle passing through the double slit occurs, accompanied by the collapse of the particle state. However, a measurement is not required for the collapse of the state to occur.

## Data Availability

The plots of figures were generated using Matlab program that the authors will make available on the first author’s website.

## Glossary

List of Main Symbols (in Order of Appearance)

**Section 2**	
*x*	3D position variable or operator.
*k*	spatial frequency variable (the Fourier transform variable of the *x* variable).
*p*	momentum variable or operator, conjugate pair to *x*
|ψ〉 and ρ=|ψ〉〈ψ|	QM state and QM density operator associated with a QM state.
$|\psi_{t}\rangle =e^{-i\frac{H}{\hbar }t}|\psi \rangle$ and $\rho_{t}=e^{-i\frac{H}{\hbar }t}\;\rho e^{i\frac{H}{\hbar }t}$	Time evolution of a QM state and of a density operator
$\psi (x,t)=\langle x|\psi_{t}\rangle$ and $\phi (p)=\langle p|\psi_{t}\rangle$	QM wave functions in each coordinate of phase space (x,p)
Entropy	coordinate-entropy in phase space
$S_{r},S_{p},S_{k}$	coordinate-entropy components for position *x*, momentum *p*, and spatial frequency *k*, respectively.
Ψ(x,t) and Φ(k,t)	QFT operators in position and spatial frequency domains, respectively.
$\rho_{x}^{\mathrm{QFT}}\left(x,t\right)=\Psi^{†}\left(x,t\right)\Psi \left(x,t\right)$ and $\rho_{k}^{\mathrm{QFT}}\left(k,t\right)=\Phi^{†}\left(k,t\right)\Phi \left(k,t\right)$	QFT probability density operators in position and spatial frequency domains, respectively.
$|n_{q_{1}},n_{q_{2}},,\cdots ,n_{q_{i}},\cdots n_{q_{K}}\rangle$	Fock states with occupation number, where $n_{q_{i}}$ is the number of particles in a QM state $|q_{i}\rangle$.
$|\mathrm{state}\rangle =\sum_{m}\alpha_{m}|n_{q_{1}},n_{q_{2}},\cdots ,n_{q_{i}},\cdots \rangle$	a QFT state in a Fock space where *m* is an index over configurations of a Fock state, $\alpha_{m}\in C$, and $1=\sum_{m}{\left|\alpha_{m}\right|}^{2}$.
Below are the same symbols for probability density values for the x,k variables used for the QM and QFT frameworks. Context will disambiguate	$\rho_{x}\left(x,t\right)=\left\{\begin{array}{cc} \langle state|\Psi^{†}\left(x,t\right)\Psi \left(x,t\right)|state\rangle : & \mathrm{QFT}\mathrm{for}\mathrm{the}\;x\;\mathrm{variable}\\ \langle x|\psi_{t}\rangle \langle \psi_{t}||x\rangle : & \mathrm{QM}\mathrm{for}\mathrm{the}\;x\;\mathrm{variable} \end{array}\right.$
	$\rho_{k}\left(k,t\right)=\left\{\begin{array}{cc} \langle state|\Phi^{†}\left(k,t\right)\Phi \left(k,t\right)|state\rangle : & \mathrm{QFT}\mathrm{for}\mathrm{the}\;k\;\mathrm{variable}\\ \langle k||\psi_{t}\rangle \langle \psi_{t}||k\rangle : & \mathrm{QM}\mathrm{for}\mathrm{the}\;k\;\mathrm{variable} \end{array}\right.$
$\lambda_{i}>0,i=1,\cdots ,m$	probability coefficients of a mixed state made of *m* pure states.
**Section 3**	
$F:\left(x,k,t\right)\mapsto \left(x^{\prime },k^{\prime },t\right)$	a canonical transformation of coordinates
$J_{F}\left(x,k,t\right)$ to be the Jacobian	
Given a QFT solution Ψ(x,t)	
$\Psi^{C}\left(x,t\right)=C{\bar{\Psi }}^{T}\left(x,t\right)$	Charge Conjugation satisfying
$C\gamma^{\mu }C^{-1}=-\gamma^{\mu T}$, and in the standard representation $C=i\gamma^{2}\gamma^{0}$ up to a phase.	
$\Psi^{P}\left(-x,t\right)=P\Psi \left(-x,t\right)$	Parity Change, so $P=\gamma^{0}$
$\Psi^{T}\left(x,-t\right)=T\Psi^{*}\left(x,-t\right)$	Time Reversal, carried by the operator $T=T\hat{K}$, where $\hat{K}$ applies conjugation. In the standard representation $T=i\gamma^{1}\gamma^{3}$, up to a phase.

$\psi^{\mathrm{CPT}}\left(-x,-t\right)=CPT{\bar{\psi }}^{T}\left(-x,-t\right)$	Charge Conjugation, Parity Change, and Time Reversal.
**Section 5**	
$U\left(t\right)=e^{-i\frac{H}{\hbar }t}$	evolution operator for Hamiltonian *H*
$\left(\langle x|\psi_{0}\rangle ,\;\langle k|\psi_{0}\rangle ,\right)$	initial QM condition for the unitary evolution
$\left(\Psi_{0}=\Psi \left(x,0\right),\;\Phi_{0}=\Phi \left(k,0\right)\right)$	initial QFT condition for the unitary evolution
QCurve	
C	The set of QCurves for which the entropy is a constant
I	The set of QCurves for which the entropy is increasing, but it is not a constant
D	The set of QCurves for which the entropy is decreasing, but it is not a constant
O	The set of oscillating QCurves, with the entropy strictly increasing in some subinterval of [0,δt] and strictly decreasing in another subinterval of [0,δt]
$N\left(x|c,\Sigma \right)$	Normal distribution for variable *x*, centered in *c*, and with covariance Σ
$\alpha_{k}\left(t\right)$	Coefficients of expansion of a QM state $|\psi_{t}\rangle$ into the energy eigenstates $|\psi_{E_{k}}\rangle$
**Section 6**	
$A^{i}\left(k\right)$	Electromagnetic potential 3D components, i=1,2,3, in spatial frequency space
$a_{\lambda }\left(k\right)$	annihilation operator per polarization λ=1,2, in spatial frequency space
$a_{\lambda }^{†}\left(k\right)$	creation operator per polarization λ=1,2, in spatial frequency space
$\epsilon_{\lambda }^{i}\left(k\right)$	polarization 3D orientation components, i−1,2,3, per polarization λ=1,2
